# Evaluation des Trabecular Bone Score (TBS) in der täglichen Praxis bei Patienten mit entzündlich rheumatischen und nichtentzündlichen Erkrankungen: Korrelation mit konventioneller Knochendichtemessung und Prävalenz von Wirbelkörperfrakturen

**DOI:** 10.1007/s00393-020-00764-9

**Published:** 2020-03-11

**Authors:** B. Buehring, J. Thomas, T. Wittkämper, X. Baraliakos, J. Braun

**Affiliations:** 1 Rheumazentrum Ruhrgebiet, Ruhr Universität Bochum, Claudiusstr. 45, 44649 Herne, Deutschland; 2 Radiologie Herne, Herne, Deutschland

**Keywords:** Rheumatoide Arthritis, Osteoporose, Wirbelkörperfraktur, Knochendichte, DXA, Rheumatoid Arthritis, Osteoporosis, Vertebral Fracture, Bone Mineral Density, DXA

## Abstract

**Hintergrund:**

Osteoporosebedingte Frakturen sind bei Patienten mit rheumatoider Arthritis (RA) häufig. Die Messung der Knochenmineraldichte (KDM) mit der Dual-Energie-Röntgenabsorptionsmessung (DXA) allein sagt das Frakturrisiko nur begrenzt voraus. Der Trabecular Bone Score (TBS) ist ein Surrogatmarker für die trabekuläre Mikroarchitektur des Knochens, der das Frakturrisiko unabhängig von der KDM vorhersagen kann.

**Ziel:**

Ermittlung der Prävalenz von KDM, TBS und osteoporotisch bedingten Wirbelkörperbrüchen („vertebral fractures“ [VF]) bei Patienten mit RA im Vergleich zu Kontrollen mit nichtentzündlichen Muskel-Skelett-Erkrankungen (MSK).

**Methoden:**

Die Daten von Patienten mit von Rheumatologen diagnostizierter RA und verfügbaren TBS- und DXA-Messungen, die in unserem Krankenhaus von 2006 bis 2014 erhoben wurden, wurden retrospektiv analysiert. Den RA-Patienten wurden Kontrollen mit nichtentzündlichen MSK zugeordnet. Eine „reduzierte Knochengesundheit“ wurde definiert als ein T‑Score <−1,0 und/oder ein TBS-Wert <−1,31. Statistische Vergleiche wurden mit dem Mann-Whitney- und dem Wilcoxon-Test durchgeführt.

**Ergebnisse:**

Es wurden 143 Patienten mit RA (Alter 72,1 ± 11,1 Jahre, 72 % weiblich) und 106 Kontrollen (Alter 69,6 ± 12,6 Jahre, 75 % weiblich) eingeschlossen. RA-Patienten hatten häufiger eine erniedrigte KDM (*n* = 102; 71,3 %) und einen erniedrigen TBS-Wert (*n* = 125; 87,4 %) als die Kontrollen (*n* = 63; 59,4 % und *n* = 79; 74,5 %, *p* = 0,049 und *p* = 0,009). RA-Patienten hatten mehr VF (*n* = 52, 36,4 %) als Kontrollen (*n* = 24, 22,6 %, *p* = 0,02). Insgesamt hatten 20 Patienten mit VF (26,3 %) eine normale Wirbelsäulen-KDM und 9 (11,8 %) auch eine normale Hüft-KDM. Bei Patienten mit VF war die Kombination eines niedrigen TBS bei normaler WS-KDM häufiger als ein normaler TBS bei niedriger WS-KDM (*p* = 0,008 für RA, *p* = 0,025 für Kontrollen).

**Diskussion:**

VF treten bei Patienten mit normaler KDM auf. Bei Patienten mit VF wurde eine niedrige TBS bei normaler Wirbelsäulen-KDM häufiger gefunden als eine normale TBS bei niedriger Wirbelsäulen-KDM. Die Messung des TBS scheint für die Erkennung eines erhöhten Frakturrisikos bei RA-Patienten mit normaler WS-KDM nützlich zu sein.

## Abstract (en)

**Background:**

Osteoporosis-related fractures are common in patients with rheumatoid arthritis (RA). Bone mineral density (BMD) measurements using dual-energy x‑ray absorptiometry (DXA) alone has only a limited value for predicting the risk of fractures. The trabecular bone score (TBS) is a surrogate parameter for trabecular microarchitecture of bone and a predictor of fracture risk independent of BMD.

**Aim:**

To examine the prevalence of BMD, TBS and osteoporosis-related vertebral fractures (VF) in patients with RA in comparison to controls with non-inflammatory musculoskeletal diseases.

**Methods:**

Data from patients with RA diagnosed by a rheumatologist and with TBS and DXA measurements, who were assessed in this hospital between 2006 and 2014 were retrospectively analyzed. The RA patients were matched with controls with non-inflammatory musculoskeletal diseases. “Reduced bone health” was defined as a T‑score <−1.0 and/or a TBS value <−1.31. Statistical analyses were carried out using the Mann-Whitney test and the Wilcoxon test.

**Results:**

Data from 143 patients with RA (age 72.1 ± 11.1 years, 72% female) and 106 controls (age 69.6 ± 12.6 years, 75% female) were included. The RA patients more frequently had low BMD (*n* = 102, 71.3%) and low TBS values (*n* = 125, 87.4%) compared to controls (*n* = 63, 59.4% and *n* = 79, 74.5%, *p* = 0.049 and *p* = 0.009, respectively). The RA patients had more VF (*n* = 52, 36.4%) than controls (*n* = 24, 22.6%, *p* = 0.02). A total of 20 patients with VF (26.3%) had normal lumbar spine BMD and 9 (11.8%) also had a normal hip BMD. In patients with VF the combination of low TBS with normal spine BMD was more common than a normal TBS and low spine BMD (*p* = 0.008 for patients with RA, *p* = 0.025 for controls).

**Discussion:**

It is known that VF can occur in patients with normal BMD. In patients with VF, a low TBS with normal spine BMD is found more frequently than normal TBS and low spine BMD. These results suggest that measurement of the TBS has the potential to be a useful tool to detect increased fracture risk in patients with RA and normal spine BMD.

Die Osteoporose ist eine systemische Skeletterkrankung, die durch niedrige Knochenmasse und -qualität charakterisiert ist [1, 2]. Folgen der Knochenfragilität sind osteoporotische Frakturen, die wegen der damit verbundenen Schmerzen, eingeschränkter Mobilität, geringerer Alltags- und Arbeitsfähigkeit und erhöhter Mortalität eine erhebliche klinische Bedeutung haben. Osteoporotische Frakturen verursachen erhebliche Kosten für alle Gesundheitssysteme. In der Allgemeinbevölkerung ist Osteoporose (nach WHO-Definition, s. unten) häufig, und jede 2. Frau und jeder 4. bis 5. Mann erleidet in Folge ihrer eine osteoporotische Fraktur [1, 3, 4]. Neben diesen primären Formen der Osteoporose (postmenopausale und altersbedingte Osteoporose) gibt es Bevölkerungs- und Patientengruppen, die ein noch deutlich höheres Risiko für osteoporotische Frakturen haben – z. B. Patienten mit entzündlich rheumatischen Erkrankungen [5–7]. So ist das Risiko für Hüft- und Wirbelkörperfrakturen bei Patienten mit rheumatoider Arthritis (RA) verdoppelt – und das ist unabhängig von der Einnahme von Glukokortikoiden [8]. Eine wirksame antientzündliche Therapie der rheumatischen Entzündung kann den Knochenverlust aber trotz der Einnahme von Glukokortikoiden aufhalten [9, 10]. Pathophysiologisch führt die chronische Entzündung über Zytokin-geleitete Mechanismen zu erhöhter Osteoklastenaktivität und Knochenresorption [5, 7, 11].

Mit Verbreitung der Dual-Energie-Röntgenabsorptionsmessung (DXA) vor mehr als 30 Jahren wurde es möglich, den Verlust an Knochenmasse bzw. Knochendichte (KD) zu quantifizieren. Schon frühe WHO-Definitionen der Osteoporose haben sich daher weitgehend auf eine niedrige Knochendichte gestützt. Im Jahr 1994 wurde die Diagnose Osteoporose bei Vorliegen eines T‑Werts von ≤−2,5 etabliert [12, 13]. Die individuelle Risikoabschätzung einer Fraktur geht aber über die Messung der Knochendichte (KDM) hinaus. So haben Langzeitstudien gezeigt, dass mehr als 50 % der osteoporotischen Frakturen oberhalb eines T‑Wertes von −2,5 auftraten [1, 9, 13–15]. Deshalb wurde zur Vorhersage von Frakturen ein Risikorechner entwickelt, um das individuelle Frakturrisiko von Patienten zu bestimmen [16, 17]. Das mittlerweile weltweit am meisten hierfür benutze Instrument ist der 2008 veröffentlichte Fracture Risk Assessment Score (FRAX) – ein Rechentool, durch den eine individuelle Abschätzung des 10-Jahres-Risikos für das Erleiden einer Hüftfraktur oder einer sog. „major fracture“ (Wirbelkörperfraktur, distale Unterarmfraktur, proximale Humerus- und Femurfraktur) erfolgen kann. Die Berechnung mithilfe des Scores basiert auf 11 Risikofaktoren sowie auf der mittels DXA gemessenen Knochendichte am Schenkelhals. Zu diesen Risikofaktoren gehören sowohl die RA als auch die Therapie mit Glukokortikoiden [1, 13, 18]. Der FRAX-Rechner ist aber nicht das einzige Werkzeug zur Bestimmung des Frakturrisikos [9]. Der Dachverband Osteologie e. V. (DVO) hat ebenfalls ein Modell zur Vorhersage von Frakturen entwickelt. Hier ist nicht nur die RA ein Risikofaktor, sondern auch andere rheumatische Erkrankungen wie die axiale Spondyloarthritis oder der systemische Lupus erythematodes (Dachverband Osteologie e. V.; www.dv-osteologie.org/dvo_leitlinien/dvo-leitlinie-2017). Bei Patienten mit entzündlich rheumatischen Erkrankungen und/oder höheren Glukokortikoiddosierungen treten osteoporotische Frakturen im Vergleich zur postmenopausalen oder altersbedingten Osteoporose schon bei T‑Werten >−2,5 (Osteopenie) auf [8, 19–21].

Unabhängig von den klinischen Risikofaktoren bleibt die durch DXA bestimmte KDM aber sowohl zur Diagnosestellung als auch zur Einschätzung des Frakturrisikos ein essenzielles Werkzeug. Die DXA kann aber nicht nur für die KDM benutzt werden, sondern auch für die Bestimmung anderer für das Frakturrisiko wichtiger Parameter wie das Vorliegen von (auch asymptomatischen) Wirbelkörperfrakturen, zur Abklärung einer Sarkopenie und auch für die Knochenmikroarchitektur in Wirbelkörpern [13]. Diese Methode wird als Trabecular Bone Score (TBS) bezeichnet. Der TBS ist ein in den letzten Jahren entwickelter Knochenstrukturparameter, welcher zunehmend Anwendung im klinischen Alltag findet. Der TBS ist ein Parameter für die die Bestimmung der Qualität der Mikroarchitektur des Knochens, was eine bessere Aussage über die Knochenstabilität ergeben soll. Berechnet wird der Score anhand von statistischen Graustufenmodellen, die auf der Grundlage von DXA-Messungen an der lumbalen Wirbelsäule angefertigt werden. Ähnlich wie bei der DXA werden die unterschiedlichen Absorptionseigenschaften von Röntgenstrahlen in verschiedenen Gewebestrukturen erfasst. Dafür werden 3‑dimensionale Gewebe in eine 2‑dimensionale Projektion mit verschiedenen Graustufen transformiert. Die Software quantifiziert dann die lokale Variation in den Graustufen von benachbarten Pixeln und berechnet daraus den TBS. Ein osteoporotischer Wirbelkörper mit einer hohen Variation hat statistisch sehr röntgenarme (wenig/kein Knochen) Bereiche und nur vereinzelte röntgendichte (Knochentrabekel) Bereiche. Ein gesunder Wirbelkörper mit dichter Trabekelstruktur hat eine homogenere Knochendichteverteilung mit weniger Variation [13, 22–25]. Bisher gibt es noch keine allgemeingültigen und verbindlichen Grenzwerte für die Ergebnisse von TBS-Messungen. Zur Einteilung und besseren Vergleichbarkeit mit DXA-Messungen wurde im Rahmen einer großen Metaanalyse 2016 ein Hoch‑, Mittel- und Niedrigrisikobereich definiert [26, 27]. Im Englischen spricht man dementsprechend von normal, „partially degraded“ und „degraded“, was die abgebaute oder geschädigte Mikroarchitektur des Knochens beschreibt. Das bedeutet auch, dass je höher der TBS Wert ist, umso besser ist die Wirbelkörpermikroarchitektur. Dank dieser Methode lässt sich also anhand einer durchgeführten KDM der lumbalen Wirbelsäule ohne eine zusätzliche Messung (und damit ohne zusätzliche Strahlenbelastung) über eine Software eine Aussage über die „Knochenqualität“ machen. Bei postmenopausalen Frauen und Männern über 50 Jahre wurden anhand von Daten aus prospektiven und Zulassungsstudien der diagnostische Nutzen und auch die von der Knochendichte unabhängige, prognostische Aussagekraft des TBS für osteoporotische Frakturen bereits nachgewiesen [27–37]. Erste Studien haben dies auch bei Patienten unter Glukokortikoidtherapie und/oder bei chronisch entzündlichen Erkrankungen gezeigt [38–46].

Inwieweit der Score auch bei Real-world-Patienten mit RA von Vorteil ist, ist bislang noch nicht endgültig geklärt. In der vorliegenden Arbeit wurde deshalb untersucht, inwieweit es Unterschiede der mit TBS erzielten Ergebnisse im Vergleich zur einfachen KDM zwischen Patienten mit RA und Patienten ohne entzündlich rheumatische Erkrankungen gibt. Als sekundäre Fragestellung wurde untersucht, inwieweit Patienten mit einer osteoporotischen Fraktur einen niedrigeren TBS haben als Patienten ohne Fraktur und ob sich die Ergebnisse des TBS von den KDM-Werten von Patienten mit einer oder mehreren osteoporotischen Frakturen unterscheiden.

## Methoden

### Patientenrekrutierung

Für diese retrospektive Fall-Kontroll-Studie wurden über das Klinikinformationssystem des Rheumazentrums Ruhrgebiet in den Jahren 2016 und 2017 Patienten rausgesucht, die im Zeitraum von Ende 2006 bis 2014 (also ca. 8 Jahre) eine radiologische Wirbelkörperfraktur hatten und zusätzlich eine DXA mit TBS-Messung in einem Zeitraum von ±1 Jahr. Aus diesem Kollektiv wurden dann Patienten mit RA und Patienten ohne entzündlich rheumatische Erkrankungen ausgewählt und diesen in Alter (±1 Jahr) und Geschlecht gleiche Patienten zugeordnet.

### Bestimmung der Knochendichte und des Trabecular Bone Score

Die DXA-Messungen wurde mit einem GE/Lunar Prodigy Densitometer, wie in den aktuellen DVO-Leitlinien empfohlen, an Lendenwirbelsäule (LWS) und Hüften durchgeführt und die Ergebnisse mit der GE/Lunar Software „enCore Version 16“ (GE/Lunar, Madison, WI, USA) ausgewertet. Zur Bewertung der Knochendichte wurden die LWS (Bereich LWK1 bis LWK4) und Hüften (Schenkelhals und gesamter proximaler Femur) ausgewählt. Zur Berechnung des TBS-Wertes wurde die installierte Software „TBS iNsight®“ der Firma Medi-Maps (Genf, Schweiz) verwendet. Anhand der gemessenen T‑Scores wurden die Patienten als „normale“ Knochendichte, *Osteopenie* oder *Osteoporose* gemäß den WHO-Empfehlungen klassifiziert. Dabei wurden T‑Scores von −1,0 oder besser als *normal *kategorisiert, T‑Scores von −1,1 bis −2,4 als *Osteopenie* und T‑Scores von −2,5 als *Osteoporose* bewertet. Für einige Analysen wurden die Patienten in eine Gruppe mit *pathologischer KD* (T-Score ≤−1,0) und in eine mit normaler KD (T-Scores −1,0 oder besser) aufgeteilt, um eine dichotome Analyse durchführen zu können. Ähnlich wurde mit den TBS-Werten verfahren [27]. Patienten mit einem TBS-Wert von 1,23 oder niedriger wurden als *Mikroarchitektur abgebaut* („degraded“) klassifiziert, Patienten mit Werten zwischen 1,24 und 1,31 als *teilweise abgebaut *und Patienten mit Werten von 1,31 oder besser als *normal* klassifiziert. Die dichotome Einteilung *pathologischer TBS/normaler TBS* wurde über einen Schwellenwert von 1,31 definiert. Da der TBS zurzeit nur an der Wirbelsäule verfügbar ist, wurden für die Einteilung der Knochendichte auch nur die Ergebnisse der lumbalen Wirbelsäule zum Vergleich herangezogen.

### Statistische Analysen

Der Vergleich zwischen den beiden untersuchten Gruppen wurde für kontinuierliche Variablen mit dem Mann-Whitney-Test und für binäre Variablen mit dem Wilcoxon-Test durchgeführt. Die statistische Datenanalyse erfolgte mit dem Statistikprogramm SPSS, Version 23 (SPSS, IBM, Armonk, NY, USA).

## Ergebnisse

### Deskriptive Darstellung der untersuchten Gruppen

Das Untersuchungskollektiv umfasste 143 Patienten mit RA, davon waren 103 weiblichen Geschlechts (72 %). Daten zur Dauer der Erkrankung lagen bei 112 der 143 RA-Patienten vor. Die durchschnittliche Erkrankungsdauer war 7,5 Jahre (Standardabweichung ±10,3 Jahre). In dieser Gruppe hatten 52 Personen eine osteoporotische Wirbelkörperfraktur (36,4 %). Patienten mit Wirbelkörperfraktur hatten eine deutlich längere Krankheitsdauer (12 Jahre) als Patienten ohne Fraktur (4 Jahre, *p* = 0,0002). Die Kontrollgruppe umfasste 106 Patienten ohne entzündlich rheumatische Erkrankung (degeneratives Wirbelsäulensyndrom, Osteoarthrose, Fibromyalgiesyndrom), davon waren 79 Frauen (74,5 %). Bei 24 Patienten lag eine osteoporotische Wirbelkörperfraktur vor (22,6 %). Die Patienten mit RA hatten häufiger Frakturen als die Kontrollen (*p* = 0,02). In der RA-Gruppe hatten die Patienten im Durchschnitt 1,9 Wirbelkörperfrakturen (Standardabweichung ±1,8), in der Kontrollgruppe waren es 1,5 Wirbelkörperfrakturen (Standardabweichung ±0,8, *p* = 0,36). Frauen waren im Mittel kleiner und leichter als Männer (1,58/1,73 m, *p* < 0,0001 und 70/81 kg, *p* = 0,086). Die demografischen Daten sind in Tab. 1 und 2 zusammengefasst. Die Unterschiede zwischen den Gruppen in Alter, Größe und Gewicht waren statistisch nicht signifikant.

**Table 1:** 

Parameter Einheit	Alter Jahre	Geschlecht % weiblich	Größe cm	Gewicht kg	Fraktur %
Mittelwert bzw. %‑Wert	72,1	72	163,4	74,9	36,4
Standardabweichung	11,1	n.a.	9,8	16,1	n.a.

**Table 2:** 

Parameter Einheit	Alter Jahre	Geschlecht % weiblich	Größe cm	Gewicht kg	Fraktur %
Mittelwert bzw. %‑Wert	69,6	74,5	164,3	76,5	22,6
Standardabweichung	12,6	n.a.	8,6	17,0	n.a.

### Analyse der Knochendichte-T-Scores und der TBS-Werte

Sowohl der konventionelle T‑Score als auch der TBS an LWS und Hüften waren in der Untersuchungsgruppe numerisch niedriger als in der Kontrollgruppe. Im statistischen Vergleich der Mittelwerte zeigte sich aber nur ein signifikanter Unterschied für den T‑Score an der rechten Hüfte. Die Ergebnisse der konventionellen KDM- und die TBS-Werte sind für beide Gruppen in Tab. 3 dargestellt.

**Table 3:** 

	RA-Patienten		Kontrollen		*p*-Wert
Parameter	Mittelwert	SD	Mittelwert	SD	
T‑Score LWS	−0,9	1,7	−0,6	1,7	0,16
T‑Score Hüfte links	−1,2	1,2	−0,9	1,4	0,1
T‑Score Hüfte rechts	−1,3	1,2	−1,0	1,3	0,04
TBS-LWS	1,170	0,128	1,199	0,124	0,146

*RA* rheumatoide Arthritis, *TBS* Trabecular Bone Score, *LWS* Lendenwirbelsäule, *SD* Standardabweichung

### Klassifizierung nach LWS-T-Scores und TBS-Werten

Pathologische Werte, wie in den Methoden definiert, lagen bei den RA-Patienten häufiger vor als in der Kontrollgruppe: 71,3 % vs. 57,5 % für pathologische T‑Scores (*p* = 0,05) und 87,5 % vs. 74,5 % für pathologische TBS-Werte (*p* = 0,009). Daraufhin wurde untersucht, bei wie vielen Patienten pathologische TBS-Werte vorlagen, während die konventionellen KDM-Werte noch normal waren und umgekehrt. Pathologische TBS-Werte bei normaler mit DXA gemessener KDM lagen in beiden Gruppen bei >40 % der Untersuchten vor – der umgekehrte Fall dagegen in nur <1 % der Fälle. In der Kategorie TBS und DXA normal lag der Prozentsatz bei den RA-Patienten mit 7,7 % der Patienten niedriger als in der Kontrollgruppe (*p* = 0,005) (Tab. 4).

**Table 4:** 

Parameter	RA-Patienten (%)	Kontrollen (%)
TBS *abgebaut* und KD *Osteoporose*	26 (18,2)	10 (9,4)
TBS und KD *pathologisch*	114 (79,7)	74 (69,8)
TBS *normal* und KD *Osteoporose*	1 (0,7)	1 (0,9)
TBS *normal* und KD *pathologisch*	8 (5,6)	5 (4,7)
TBS *pathologisch* und KD *normal*	65 (45,5)	47 (44,3)
TBS *abgebaut* und KD *normal*	45 (31,5)	33 (31,1)
TBS und KD *normal*	11 (7,7)	21 (19,8)

*TBS* Trabecular Bone Score, *KD* Knochendichte (DXA), die Definitionen für *normal, abgebaut, pathologisch und Osteoporose sind ansonsten im Methodenteil gegeben*

### Ergebnisse, bezogen auf das Vorliegen einer Wirbelkörperfraktur

Patienten mit Wirbelkörperfrakturen hatten unabhängig von der Gruppenzugehörigkeit eine niedrigere KD an der LWS und den Hüften und auch niedrigere TBS-Werte (*p*-Werte in beiden Gruppen bei allen Parametern unter 0,001). Im Vergleich der einzelnen T‑Scores bzw. TBS-Werte ergab sich bei Teilnehmern mit und ohne Fraktur zwischen RA-Patienten und Kontrollen kein signifikanter Unterschied. Bei 27 % der RA-Patienten und bei 25 % der Kontrollen mit osteoporotischen Wirbelkörperfrakturen fand sich eine normale KD an der LWS.

Bei wie vielen Patienten mit schon eingetretener Fraktur ein pathologisch veränderter TBS-Wert (<1,31) vorlag, während die KD-Werte der LWS noch normal waren, zeigen Tab. 5 und 6. Unter den RA-Patienten hatten 17 (32,7 %) eine normale KD an der LWS, während der TBS-Wert pathologisch war. Umgekehrt war dies nur bei 2 RA-Patienten (3,8 %) der Fall (*p* = 0,001). Bei den Kontrollen hatten 20,8 % eine normale LWS-KD bei pathologischem TBS-Wert, während der umgekehrte Fall gar nicht vorkam (*p* = 0,025).

**Table 5:** 

		TBS	
		*Pathologisch *(%)	*Normal *(%)
KD LWS	*Pathologisch*	45 (86,5)	2 (3,8)
	*Normal*	17 (32,7)	2 (3,8)

*TBS* Trabecular Bone Score, *KD* Knochendichte (DXA), *LWS* Lendenwirbelsäule; die Definitionen für *normal* und *pathologisch sind im Methodenteil gegeben*

**Table 6:** 

		TBS	
		*Pathologisch *(%)	*Normal *(%)
KD LWS	*Pathologisch*	23 (95,8)	0
	*Normal*	5 (20,8)	1 (4,2)

*TBS* Trabecular Bone Score, *KD* Knochendichte (DXA), *LWS* Lendenwirbelsäule; die Definitionen für *normal* und *pathologisch sind im Methodenteil gegeben*

## Diskussion

In dieser Real-world-Fall-Kontroll-Studie wurde bestätigt, dass sowohl Patienten mit RA als auch nichtentzündliche Kontrollpatienten mit osteoporotischer Wirbelkörperfraktur niedrigere T‑Scores und TBS-Werte hatten als solche ohne Fraktur. So hatten etwa 25 % aller Teilnehmer mit vorhandener Wirbelkörperfraktur eine normale Knochendichte an der LWS. Auch wenn der TBS-Wert bei Patienten mit RA in dieser Studie nicht niedriger war als bei Patienten ohne entzündlich rheumatische Erkrankung, hatten RA-Patienten doch häufiger pathologische TBS-Werte als die Kontrollen. Insgesamt hatten viele Patienten einen normalen T‑Score, während der TBS-Wert schon im pathologischen Bereich war. Dieses Ergebnis hat klinische Relevanz, weil man bei einer normalen Knochendichte eher ein niedriges Frakturrisiko annimmt, obwohl das tatsächliche Frakturrisiko schon nachweisbar erhöht ist. Ein pathologischer TBS-Wert könnte als weiterer Messparameter zusätzlich zu klinischen Risikofaktoren (z. B. Kortison, rheumatologische Grunderkrankung, prävalente Frakturen) hinzugezogen werden und früher Anlass zu therapeutischen Interventionen geben als eben die konventionell gemessene Knochendichte. Da die TBS-Bestimmung im Rahmen der üblichen DXA-Messung der Knochendichte erfolgt, sind keine weitere Bildgebung und damit auch keine zusätzliche Strahlenexposition erforderlich. Fairerweise muss hier aber auch gesagt werden, dass die (einmalige) Anschaffung der TBS-Software zusätzliche Kosten erzeugt.

Dass die Kombination aus pathologischem TBS-Wert bei normaler konventionell gemessener Knochendichte so viel häufiger auftritt als umgekehrt, lässt sich aus unserer Sicht hauptsächlich durch 2 Faktoren erklären. Erstens gibt es in der Wirbelsäule sehr häufig degenerative Veränderungen, die die konventionell gemessene LWS-Knochendichte falsch hoch erscheinen lassen können [47, 48]. Dies liegt an der DXA-Messtechnik, bei der der Wirbelkörper als 3‑dimensionale Struktur auf eine 2‑dimensionale Fläche reduziert wird. Das beinhaltet eben auch, dass degenerative Veränderungen, wie z. B. Spondylophyten, Facettengelenkarthrosen, Längsbandverkalkung und DISH im Bereich der LWS, zu einer artifiziellen Erhöhung der Knochendichte führen können, aber eben keine reduzierende Wirkung auf die Kräfte haben, die zu Wirbelkörperfrakturen führen. In einer zusätzlichen Analyse, in der sowohl die T‑Scores der LWS als auch die der Hüfte eingingen, sank die Zahl von Teilnehmern mit osteoporotischer Wirbelkörperfraktur, normaler Knochendichte und pathologischem TBS von 27 % auf 13,5 % bei RA-Patienten und von 25 % auf 8 % bei den Kontrollen – ein Zeichen dafür, wie wichtig es ist, auch die Knochendichte an der Hüfte zu messen. Dass es aber selbst nach Hinzunehmen der Knochendichte an der Hüfte immer noch ca. 10 % Patienten mit osteoporotischen Wirbelkörperfrakturen gibt, die pathologische TBS-Werte und eine normale Knochendichte aufweisen, zeigt, dass es nicht nur um Effekte der konventionellen DXA-Messung geht. So lässt sich pathophysiologisch als zweiten Grund anführen, dass es Knochendichte-unabhängige Faktoren gibt, die das Frakturrisiko erhöhen. Die trabekuläre Mikroarchitektur, für die der TBS ein Surrogatparameter ist, ist ein solcher Faktor. Die Verschlechterung der Knochenarchitektur (charakterisiert durch eine kleinere Anzahl an Trabekeln im spongiösen Knochen, einem vergrößerten Abstand zwischen den Trabekeln, sowie einem Rückgang der Konnektivität zwischen den Trabekeln) führt dazu, dass einwirkende Kräfte schneller zur Fraktur führen [25, 30, 35, 49, 50].

Seit die TBS-Technik 2008 implementiert wurde, haben viele Studien den Nutzen dieses Parameters untersucht. In verschiedenen Querschnittstudien wurde dabei gezeigt, dass ein niedriger TBS-Wert mit osteoporotischen Frakturen bei postmenopausalen Frauen assoziiert ist [22, 28, 29, 34]. Auch prospektive Studien kamen zu dem Ergebnis, dass der TBS-Wert das Auftreten von Frakturen bei postmenopausalen Frauen und älteren Männern vorhersagt [31–33, 35, 51]. Eine Metaanalyse, in welcher 14 populationsbasierte Kohorten aus Nordamerika, Asien, Australien und Europa mit 17.809 Patienten untersucht wurden, kam zu dem Schluss, dass der TBS-Wert ein vom durch FRAX ermittelten Risiko unabhängiger Parameter ist, um die Wahrscheinlichkeit einer Fraktur vorherzusagen. Außerdem erlaubt eine Kombination des TBS mit dem FRAX-Score eine bessere Vorhersage für Hüft- und andere osteoporotische Frakturen. Dies gilt auch für die Kombination des TBS mit einzelnen Risikofaktoren, welche in das FRAX-Instrument einfließen [27]. Wie oben erwähnt, ist ein weiterer Vorteil des TBS gegenüber der konventionell gemessenen KDM der Wirbelsäule eine größere Unabhängigkeit von Messungsartefakten. So können degenerative Veränderungen das Ergebnis der KDM fälschlicherweise anheben. Studien belegen, dass der TBS von solchen Störeinflüssen weniger abhängig ist [26, 52].

Patienten mit RA haben grundsätzlich ein höheres Risiko, eine Fraktur zu erleiden, als die Normalbevölkerung [8, 21, 53]. Studien zeigen, dass Patienten mit RA per se ein höheres Risiko für Wirbelkörperfrakturen haben als solche mit postmenopausaler Osteoporose. Dies spiegelt sich allerdings nicht unbedingt in der gemessenen Knochendichte wider [8, 21, 54–57]. Dies liegt daran, dass die konventionell gemessene Knochendichte Veränderungen der Knochenqualität nur partiell erfasst. Bei entzündlichen Erkrankungen wie der RA wie auch unter einer systemischen Glukokortikoidtherapie kommt es im Vergleich zur postmenopausalen Osteoporose vermehrt zu einem Abbau der Mikroarchitektur und negativen Einflüssen auf die Reparaturmechanismen des Knochens („bone remodeling“), da nicht nur die Funktion der Osteoklasten (verstärkt), sondern auch der Osteozyten und Osteoblasten (verringert) beeinflusst wird [20, 47, 53, 58–61]. So frakturieren Patienten mit Glukokortikoid-induzierter Osteoporose bei gleicher Knochendichte häufiger als Patienten mit postmenopausaler Osteoporose oder sogar früher bei besseren Knochendichtewerten [21, 53]. Inwieweit der TBS als Parameter für die Mikroarchitektur bei rheumatischen Erkrankungen eine bessere Vorhersage auf Frakturereignisse treffen kann, ist aber noch nicht abschließend geklärt, da nur wenige Studien zu dieser Fragestellung vorliegen. Eine Studie aus Korea mit RA-Patientinnen kam zu dem Schluss, dass der TBS sowohl besser mit dem FRAX korreliert als die Knochendichte und auch der konventionell gemessenen Knochendichte in der Identifikation von Patienten mit Wirbelkörperfrakturen überlegen ist [36]. Ähnliche Ergebnisse wurden bei Patienten mit Polymyalgia rheumatica publiziert [45]. In einer französischen Arbeit zeigte der TBS-Wert eine bessere Vorhersage für das Auftreten von Wirbelkörperfrakturen bei Patienten mit RA an als die konventionell gemessene Knochendichte. Insbesondere scheint der TBS hilfreich zu sein, wenn diese nur ein niedriges Frakturrisiko vorhersagt [38].

Eine Stärke der hier vorliegenden Arbeit ist die große Anzahl an Patienten mit der gesicherten Diagnose einer osteoporotischen Fraktur bei Vorliegen einer RA und die ähnlich große Kontrollgruppe. Eine Limitation dieser Arbeit ist das retrospektive Studiendesign und die fehlende Adjustierung nach Glukokortikoidexposition. Eine retrospektive Fall-Kontroll-Studie wie die vorliegende erlaubt grundsätzlich keine Rückschlüsse auf eine kausale Beziehung zwischen TBS-Wert und dem Auftreten einer Fraktur. Ob in dieser Studie die niedrigeren TBS-Werte in der RA-Gruppe auf die chronisch rheumatische Entzündung oder auf Nebenwirkungen der Glukokortikoidtherapie zurückzuführen sind, lässt sich nicht näher differenzieren. Daten zu Krankheitsaktivität, Glukokortikoiddosis, DMARD- und Biologikatherapie wurden nicht dokumentiert. Daten zur Knochengesundheit wie Kalzium- und Vitamin-D-Substitution, Knochenumbauparameter oder nichtvertebrale Frakturen wurden ebenfalls nicht erfasst. Diese Limitationen erschweren es, die Ergebnisse dieser Studie zu generalisieren und Rückschlüsse zu ziehen, ob es bestimmte rheumatologische Patientenpopulationen gibt, die besonders vom Einsatz des TBS profitieren können. Bisher gibt es nur wenige Studien, die dies untersucht haben. In einer Studie war der TBS bei Patienten mit RA oder systemischer Sklerose im Vergleich zu gesunden Kontrollen signifikant niedriger. Unterschiede in der Glukokortikoiddosis konnten dies potenziell erklären [39]. Darüber hinaus konnte gezeigt werden, dass Patienten, die eine systemische Glukokortikoidtherapie erhielten, einen niedrigeren TBS-Wert im Vergleich zu Kontrollen hatten [40, 62, 63]. Da Patienten mit RA oft mit Glukokortikoiden behandelt werden, beeinflussen diese sicherlich die Knochendichte und den TBS in dieser Patientengruppe. In einer anderen Studie wurde bei RA-Patienten zwar eine Assoziation zwischen der kumulativen Glukokortikoiddosis und den TBS-Werten gefunden, nicht aber zwischen TBS und Krankheitsaktivität [37]. Größere und v. a. prospektive Studien werden nötig sein, um den Effekt von Glukokortikoiden und Krankheitsaktivität besser differenzieren zu können. Ähnliches gilt für den Einfluss von anderen Medikamenten, insbesondere der verschiedenen DMARDs und osteologischen Medikamenten. Die derzeitige Studienlage zeigt, dass sowohl antiresorptive als auch osteoanabole Therapien den Verlust an Knochendichte stoppen bzw. sogar zu einer Zunahme an Knochendichte führen. Bei konventionellen und biologischen DMARDs sind die Daten gemischt, wobei man annehmen kann, dass diese Medikamente auch einen positiven Effekt auf die Knochendichte haben. Dies lässt sich wahrscheinlich auf eine Reduktion der Entzündung zurückführen. Neuere Studien finden auch Zeichen dafür, dass Autoantikörper der RA selbst Osteoklastenaktivität beeinflussen können [7, 64, 65]. Inwiefern TBS als Methode zur Therapieüberwachung bei Osteoporose eingesetzt werden kann, ist noch nicht endgültig geklärt. Erste Studien zeigen, dass sich der TBS unter osteoanaboler Therapie verbessert, unter Bisphosphonat-Therapie sich aber nicht verändert [46, 66]. Daten zum Einfluss von DMARD-Therapie auf den TBS fehlen.

Zusammenfassend zeigt diese Studie, dass eine nicht zu vernachlässigende Anzahl von Patienten mit frakturierender Osteoporose normale Knochendichtewerte hat, besonders in der LWS-Messregion. Bei der Mehrheit dieser Patienten war der TBS-Wert schon im pathologischen Bereich, wenn die konventionell gemessenen Knochendichtewerte noch normal waren. Die Bestimmung des TBS-Wertes hat deshalb wegen der besseren Einschätzung des Frakturrisikos klinische Relevanz, besonders bei Menschen mit entzündlich rheumatischen Erkrankungen und/oder Glukokortikoidtherapie, da diese Patienten bei besseren Knochendichtewerten häufiger und schneller frakturieren. Um die prognostische Aussagekraft des TBS bei Patienten mit RA weiter zu verifizieren, sind längerfristige prospektive Studien mit größeren Patientenkollektiven erforderlich.

## References

[1] Compston JE, McClung MR, Leslie WD (2019). Osteoporosis. Lancet.

[2] Kanis JA (1994). The diagnosis of osteoporosis. J Bone Miner Res.

[3] Johnell O, Kanis JA (2006). An estimate of the worldwide prevalence and disability associated with osteoporotic fractures. Osteoporos Int.

[4] Hernlund E (2013). Osteoporosis in the European Union: medical management, epidemiology and economic burden. A report prepared in collaboration with the international osteoporosis foundation (IOF) and the European federation of pharmaceutical industry associations (EFPIA). Arch Osteoporos.

[5] Guler-Yuksel M (2018). Glucocorticoids, inflammation and bone. Calcif Tissue Int.

[6] Dischereit G, Lange U (2019). Rheumatism and bone metabolism. Orthopade.

[7] Adami G (2019). Osteoporosis in rheumatic diseases. Int J Mol Sci.

[8] van Staa TP (2006). Clinical assessment of the long-term risk of fracture in patients with rheumatoid arthritis. Arthritis Rheum.

[9] Haugeberg G (2005). Reduced loss of hand bone density with prednisolone in early rheumatoid arthritis: results from a randomized placebo-controlled trial. Arch Intern Med.

[10] Dubrovsky AM, Lim MJ, Lane NE (2018). Osteoporosis in rheumatic diseases: anti-rheumatic drugs and the skeleton. Calcif Tissue Int.

[11] Coury F, Peyruchaud O, Machuca-Gayet I (2019). Osteoimmunology of bone loss in inflammatory rheumatic diseases. Front Immunol.

[12] Faulkner KG (2005). The tale of the T-score: review and perspective. Osteoporos Int.

[13] Carey JJ, Buehring B (2018). Current imaging techniques in osteoporosis. Clin Exp Rheumatol.

[14] Schuit SC (2004). Fracture incidence and association with bone mineral density in elderly men and women: the Rotterdam study. Bone.

[15] Siris ES (2004). Bone mineral density thresholds for pharmacological intervention to prevent fractures. Arch Intern Med.

[16] Kanis JA (2007). The use of clinical risk factors enhances the performance of BMD in the prediction of hip and osteoporotic fractures in men and women. Osteoporos Int.

[17] Siris E, Delmas PD (2008). Assessment of 10-year absolute fracture risk: a new paradigm with worldwide application. Osteoporos Int.

[18] Kanis JA (2010). Development and use of FRAX in osteoporosis. Osteoporos Int.

[19] Buehring B (2013). Glucocorticoid-induced osteoporosis: an update on effects and management. J Allergy Clin Immunol.

[20] Buckley L, Humphrey MB (2018). Glucocorticoid-induced osteoporosis. N Engl J Med.

[21] Van Staa TP (2003). Bone density threshold and other predictors of vertebral fracture in patients receiving oral glucocorticoid therapy. Arthritis Rheum.

[22] Pothuaud L (2009). Evaluation of the potential use of trabecular bone score to complement bone mineral density in the diagnosis of osteoporosis: a preliminary spine BMD-matched, case-control study. J Clin Densitom.

[23] Silva BC (2014). Trabecular bone score: a noninvasive analytical method based upon the DXA image. J Bone Miner Res.

[24] Harvey NC (2015). Trabecular bone score (TBS) as a new complementary approach for osteoporosis evaluation in clinical practice. Bone.

[25] Winzenrieth R, Michelet F, Hans D (2013). Three-dimensional (3D) microarchitecture correlations with 2D projection image gray-level variations assessed by trabecular bone score using high-resolution computed tomographic acquisitions: effects of resolution and noise. J Clin Densitom.

[26] Dufour R (2013). Generation and validation of a normative, age-specific reference curve for lumbar spine trabecular bone score (TBS) in French women. Osteoporos Int.

[27] McCloskey EV (2016). A meta-analysis of trabecular bone score in fracture risk prediction and its relationship to FRAX. J Bone Miner Res.

[28] Rabier B (2010). A multicentre, retrospective case-control study assessing the role of trabecular bone score (TBS) in menopausal caucasian women with low areal bone mineral density (BMDa): analysing the odds of vertebral fracture. Bone.

[29] Winzenrieth R (2010). A retrospective case-control study assessing the role of trabecular bone score in postmenopausal caucasian women with osteopenia: analyzing the odds of vertebral fracture. Calcif Tissue Int.

[30] Hans D (2011). Correlations between trabecular bone score, measured using anteroposterior dual-energy X-ray absorptiometry acquisition, and 3-dimensional parameters of bone microarchitecture: an experimental study on human cadaver vertebrae. J Clin Densitom.

[31] Boutroy S (2013). Trabecular bone score improves fracture risk prediction in non-osteoporotic women: the OFELY study. Osteoporos Int.

[32] Briot K (2013). Added value of trabecular bone score to bone mineral density for prediction of osteoporotic fractures in postmenopausal women: the OPUS study. Bone.

[33] Iki M (2014). Trabecular bone score (TBS) predicts vertebral fractures in Japanese women over 10 years independently of bone density and prevalent vertebral deformity: the Japanese population-based osteoporosis (JPOS) cohort study. J Bone Miner Res.

[34] Krueger D (2014). Spine trabecular bone score subsequent to bone mineral density improves fracture discrimination in women. J Clin Densitom.

[35] Leslie WD (2014). Spine bone texture assessed by trabecular bone score (TBS) predicts osteoporotic fractures in men: the Manitoba bone density program. Bone.

[36] Kim D (2016). Association between trabecular bone score and risk factors for fractures in Korean female patients with rheumatoid arthritis. Mod Rheumatol.

[37] Choi YJ (2017). Trabecular bone score as a supplementary tool for the discrimination of osteoporotic fractures in postmenopausal women with rheumatoid arthritis. Medicine.

[38] Bréban S (2012). Identification of rheumatoid arthritis patients with vertebral fractures using bone mineral density and trabecular bone score. J Clin Densitom.

[39] Koumakis E (2015). Trabecular bone score in female patients with systemic sclerosis: comparison with rheumatoid arthritis and influence of glucocorticoid exposure. J Rheumatol.

[40] Paggiosi MA, Peel NF, Eastell R (2015). The impact of glucocorticoid therapy on trabecular bone score in older women. Osteoporos Int.

[41] Boussoualim K (2018). Evaluation of bone quality with trabecular bone score in active spondyloarthritis. Joint Bone Spine.

[42] Kang KY (2018). Severity of sacroiliitis and erythrocyte sedimentation rate are associated with a low trabecular bone score in young male patients with ankylosing spondylitis. J Rheumatol.

[43] Kang KY (2018). Trabecular bone score as an assessment tool to identify the risk of osteoporosis in axial spondyloarthritis: a case—control study. Baillieres Clin Rheumatol.

[44] Kang Kwi Y (2018). Associations between trabecular bone score and vertebral fractures in patients with axial spondyloarthritis. Baillieres Clin Rheumatol.

[45] Kim HA (2019). Trabecular bone score is a useful parameter for the prediction of vertebral fractures in patients with polymyalgia rheumatica. J Clin Densitom.

[46] Saag KG (2016). Trabecular bone score in patients with chronic glucocorticoid therapy-induced osteoporosis treated with alendronate or teriparatide. Arthritis Rheumatol.

[47] Buehring B (2010). Vertebral fracture assessment: impact of instrument and reader. Osteoporos Int.

[48] Krueger D (2019). DXA errors are common and reduced by use of a reporting template. J Clin Densitom.

[49] Bouxsein ML (2005). Determinants of skeletal fragility. Best Pract Res Clin Rheumatol.

[50] Bouxsein ML, Karasik D (2006). Bone geometry and skeletal fragility. Curr Osteoporos Rep.

[51] Hans D (2011). Bone microarchitecture assessed by TBS predicts osteoporotic fractures independent of bone density: the Manitoba study. J Bone Miner Res.

[52] Kolta S (2014). TBS result is not affected by lumbar spine osteoarthritis. Osteoporos Int.

[53] Roux C (2011). Osteoporosis in inflammatory joint diseases. Osteoporos Int.

[54] El Maghraoui A (2010). Prevalence and risk factors of vertebral fractures in women with rheumatoid arthritis using vertebral fracture assessment. Baillieres Clin Rheumatol.

[55] Ørstavik RE (2004). Bone health and osteoporosis: a report of the surgeon general vertebral deformities in rheumatoid arthritis.

[56] Peel NF (1995). Risk of vertebral fracture and relationship to bone mineral density in steroid treated rheumatoid arthritis. Ann Rheum Dis.

[57] Spector TD (1993). Risk of vertebral fracture in women with rheumatoid arthritis. BMJ.

[58] Chappard D (1996). Altered trabecular architecture induced by corticosteroids: a bone histomorphometric study. J Bone Miner Res.

[59] Lespessailles E (2000). Long-term corticosteroid therapy induces mild changes in trabecular bone texture. J Bone Miner Res.

[60] Walsh MC (2018). Updating osteoimmunology: regulation of bone cells by innate and adaptive immunity. Nat Rev Rheumatol.

[61] Wang T, He C (2019). TNF-alpha and IL-6: the link between immune and bone system. Curr Drug Targets.

[62] Redondo L (2018). Utilidad del trabecular bone score en la valoración del riesgo de fractura osteoporótica. Rev Clin Esp.

[63] Xue Y (2018). Lumbar spine trabecular bone score (TBS) reflects diminished bone quality in patients with diabetes mellitus and oral glucocorticoid therapy. J Clin Densitom.

[64] Schett G (2017). Autoimmunity as a trigger for structural bone damage in rheumatoid arthritis. Mod Rheumatol.

[65] Zerbini CAF (2017). Biologic therapies and bone loss in rheumatoid arthritis. Osteoporos Int.

[66] Krohn K (2019). Dual-energy X-ray absorptiometry monitoring with trabecular bone score: 2019 ISCD official position. J Clin Densitom.

